# Bis-Phenoxo-Cu^II^_2_ Complexes: Formal Aromatic Hydroxylation via Aryl-Cu^III^ Intermediate Species

**DOI:** 10.3390/molecules25204595

**Published:** 2020-10-09

**Authors:** Xavi Ribas, Raül Xifra, Xavier Fontrodona

**Affiliations:** 1 Institut de Química Computacional i Catàlisi and Departament de Química, Universitat de Girona, Campus Montilivi, E-17003 Girona, Catalonia, Spain; rxifra@cidqo.com; 2 Serveis Tècnics de Recerca (STR), Universitat de Girona, Parc Científic i Tecnològic, E-17003 Girona, Catalonia, Spain; xavier.fontrodona@udg.edu

**Keywords:** organometallic Cu^III^, C-O cross coupling, phenol synthesis, phenoxo-bridged Cu^II^ complexes, aromatic hydroxylation, copper

## Abstract

Ullmann-type copper-mediated arylC-O bond formation has attracted the attention of the catalysis and organometallic communities, although the mechanism of these copper-catalyzed coupling reactions remains a subject of debate. We have designed well-defined triazamacrocyclic-based aryl-Cu^III^ complexes as an ideal platform to study the C-heteroatom reductive elimination step with all kinds of nucleophiles, and in this work we focus our efforts on the straightforward synthesis of phenols by using H_2_O as nucleophile. Seven well-defined aryl-Cu^III^ complexes featuring different ring size and different electronic properties have been reacted with water in basic conditions to produce final bis-phenoxo-Cu^II^_2_ complexes, all of which are characterized by XRD. Mechanistic investigations indicate that the reaction takes place by an initial deprotonation of the NH group coordinated to Cu^III^ center, subsequent reductive elimination with H_2_O as nucleophile to form phenoxo products, and finally air oxidation of the Cu^I^ produced to form the final bis-phenoxo-Cu^II^_2_ complexes, whose enhanced stability acts as a thermodynamic sink and pushes the reaction forward. Furthermore, the corresponding triazamacrocyclic-Cu^I^ complexes react with O_2_ to undergo 1e^−^ oxidation to Cu^II^ and subsequent C-H activation to form aryl-Cu^III^ species, which follow the same fate towards bis-phenoxo-Cu^II^_2_ complexes. This work further highlights the ability of the triazamacrocyclic-Cu^III^ platform to undergo aryl-OH formation by reductive elimination with basic water, and also shows the facile formation of rare bis-phenoxo-Cu^II^_2_ complexes.

## 1. Introduction

Fundamental mechanistic understanding of Ullmann-type aryl-heteroatom cross-coupling chemistry is still scarce and difficult to obtain in actual catalytic systems, due to the elusive formation of very reactive intermediate species [1,2,3,4,5,6,7]. Methodological approaches consist of extensive optimization protocols to finally reach an effective method to obtain the desired reaction and performance [8,9,10], at the expense of intrinsic mechanistic understanding. Indeed, spectroscopic monitoring of the reactions is precluded by the use of high concentrated solutions and heterogeneous bases. It is proposed that complex mixtures of copper-complexes are involved, and that several mechanisms can be active in parallel. Nevertheless, one of the most accepted mechanisms involves a 2e−Cu^I^/Cu^III^ catalytic cycle via the classical oxidative addition/reductive elimination steps [7,11].

A successful strategy to overcome the problem of mechanistic understanding is the design of macrocyclic substrate scaffolds to tame the reactivity of the intermediate copper species [7,10,12]. In this manner, well-defined aryl-Cu^III^ key intermediate species have been isolated and crystallized, and their reactivity in reductive elimination processes with heteroatom nucleophiles (O, N, S, Se, P) has been widely studied [11,12,13,14,15,16,17,18,19,20,21,22,23]. In addition, upgrading to catalytic C-Heteroatom cross couplings has been proved in some of them [7,11,21]. A particularly interesting type of nucleophiles are those bearing *O*-heteroatoms, which streamline the synthesis of biaryl ethers and aryl-alkyl ethers [24,25,26], and this copper-catalyzed reactivity has proved to be very effective [8,27]. In this regard, we reported a detailed mechanistic investigation on the reactivity of well-defined triazamacrocyclic aryl-Cu^III^ species with HO-nucleophiles (HONuc = carboxylic acids, phenols and aliphatic alcohols) [26]. These reactions afforded the corresponding aryl-*O*-Nuc products under mild conditions, via a reductive elimination path.

A remarkable case of C-O coupling is the synthesis of phenol (aryl-OH), since this would imply the use of water as a nucleophile. Actually, the current synthetic methods of phenol include the classical non-metal-catalyzed transformations, such as (a) the oxidation of aryl aldehydes or aryl ketones with H_2_O_2_ (Dakin reaction) [28] and (b) the reaction of water with diazo-aryl compounds (Sandmeyer reaction) [29], and the transition metal-catalyzed transformations, such as (c) Pd-catalyzed cross coupling reactions using H_2_O [30,31] and (d) Cu-catalyzed cross coupling reactions using H_2_O [32,33], among many others [34,35,36].

**Figure 1:**
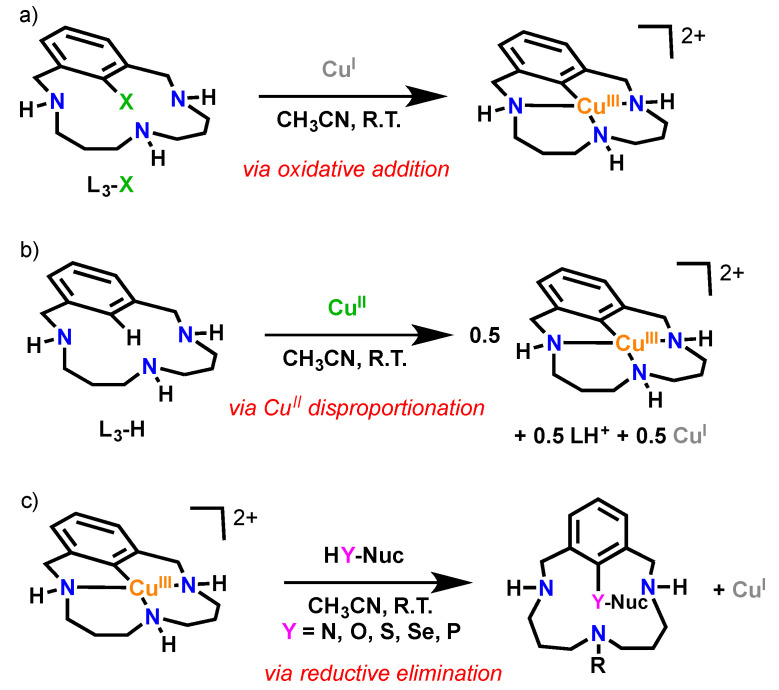
Fundamental organometallic reaction features by triazamacrocyclic ligands (L_3_-X and L_3_-H shown). (**a**) Quantitative formation of aryl-Cu^III^ complex through oxidative addition at Cu^I^ with L_3_-X (X = Cl, Br, I). (**b**) Equimolar formation of aryl-Cu^III^ complex and Cu^I^ through Cu^II^ disproportionation upon aromatic C-H activation of L_3_-H at Cu^II^. (**c**) C-Heteroatom bond formation through reductive elimination of HY-Nuc (Y N, O, S, Se, P) with the aryl-Cu^III^ complex.

In this work, we study the reactivity of well-defined triazamacrocyclic aryl-Cu^III^ species with water to evaluate the possibility to synthesize phenol products and to understand the mechanistic details of this coupling. The triazamacrocyclic aryl-Cu^III^ species can be obtained via two synthetic strategies: (1) quantitative formation via Cu^I^ oxidative addition with triazamacrocyclic aryl-X substrates (Figure 1a) [11,16,23], or (2) via C-H activation and metalation with Cu^II^ using triazamacrocyclic aryl-H substrates and further disproportionation to afford equimolar amounts of the desired aryl-Cu^III^, Cu^I^ salt and protonated substrate (Figure 1b) [16,37]. The unreported reactivity of the well-defined aryl-Cu^III^ complexes with water in basic conditions is presented in this work, leading to aryl-OH coupling species, a formal aromatic hydroxylation of arenes. The crystal structures of the final bis-phenoxo-Cu^II^_2_ complexes nicely show the effectivity of the C-O reductive elimination at Cu^III^ and the easy oxidation of the resulting Cu^I^ to bis-phenoxo-Cu^II^_2_ complexes as thermodynamic sink.

## 2. Results and Discussion

### 2.1. Aryl-Cu^III^ Complexes and Their Reactivity with Basic Water

The well-defined macrocyclic aryl-Cu^III^ complexes (**1**_L1_–**1**_L3_) used in this work were prepared following our reported protocols [11,16,23,24,37].

**Figure 2:**
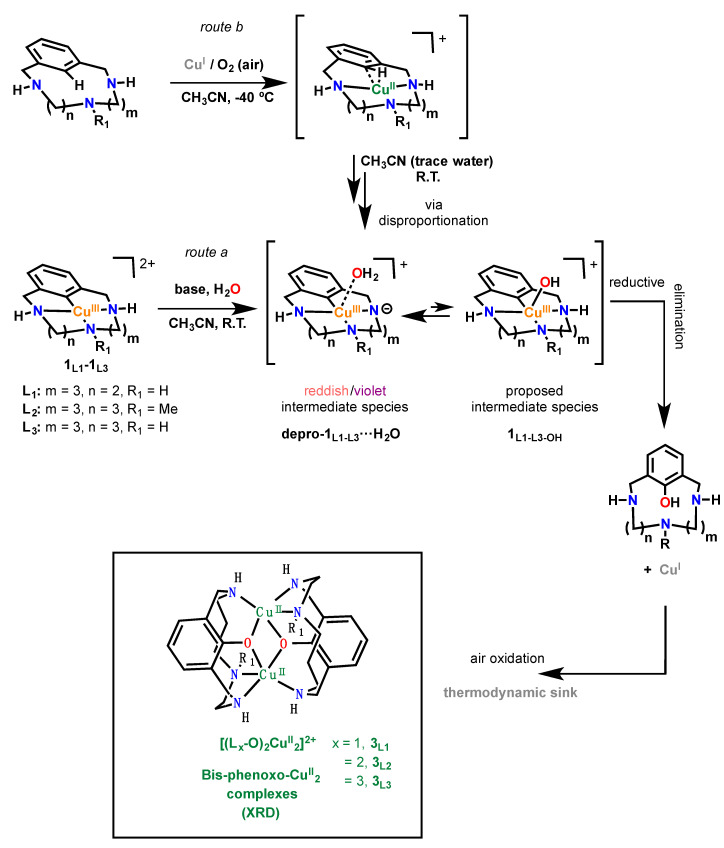
Reactivity of aryl-Cu^III^ complexes with basic water (**route a**) through initial deprotonation, axial coordination of H_2_O, internal deprotonation of water to hydroxide and reductive elimination to form the aryl-OH products and Cu^I^. Subsequent air oxidation of Cu^I^ to Cu^II^ causes the formation of the very stable bis-phenoxo-Cu^II^_2_ final complexes. On the upper part of the figure (**route b**), the same colored deprotonated aryl-Cu^III^ species can be obtained through Cu^I^/air reaction via concomitant C-H activation and disproportionation at Cu^II^. These reactions are featured with triaazamacrocyclic systems L_1_–L_3_.

Complexes [(L_x_)Cu^III^](X)_2_ (x = 1–3; X = ClO_4_^−^, OTf^−^, PF_6_^−^) dissolved in CH_3_CN react with one equivalent of aqueous KOH 1M at room temperature to give colored intermediates (Figure 2, route a). Solution acquires a red-brown (when L_1_ is used) or deep-violet (when L_2_–L_3_ is used) color, which fade to obtain final green solutions. Colored intermediates take 2–3 h to totally fade to green products. Slow diethyl ether diffusion leads to the final bis-phenoxo complexes as green crystals: [(L_1_-O)_2_Cu^II^_2_](OTf)_2_ (**3**_L1_-(OTf)_2_) in 30% isolated yield, [(L_2_-O)_2_Cu^II^_2_](X)_2_ (**3**_L2_-(X)_2_, X = OTf, ClO_4_) in 65% yield and [(L_3_-O)_2_Cu^II^_2_](PF_6_)_2_ (**3**_L3_-(PF_6_)_2_) in 60% yield.

The structures are all analogous and consist of a dimetallic Cu^II^ complex showing a N_3_O_2_ distorted trigonal bipyramidal geometry for each metal, where the two phenoxo groups are bridging and the three amine moieties belong to the two ligands featured in the structure.

### 2.2. X-ray Diffraction Analysis of the Bis-Phenoxo-Cu^II^_2_ Complexes (***3**_L1_–**3**_L3_*)

**Figure 3:**
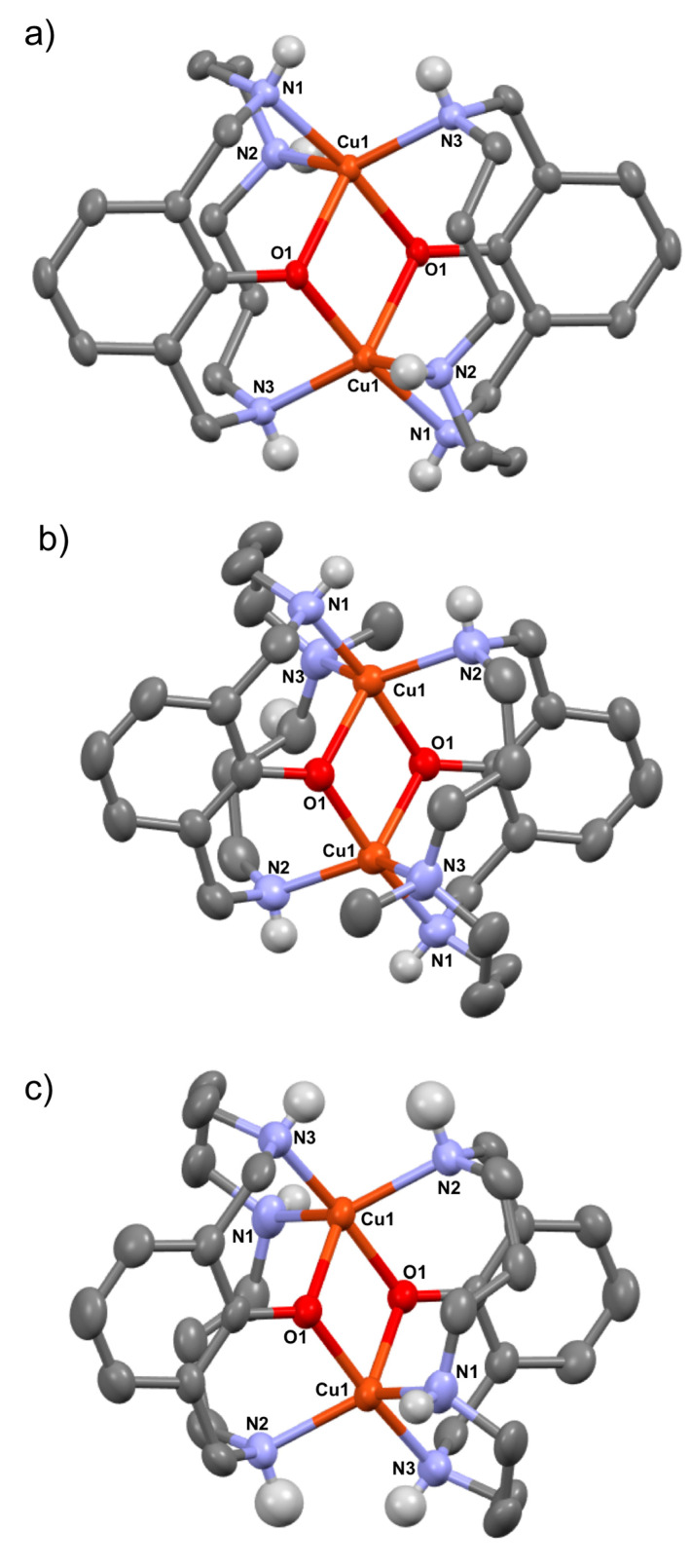
ORTEP diagrams corresponding to the cationic fragments of complexes (**a**) [(L_1_-O)_2_Cu^II^_2_](OTf)_2_ (**3**_L1_-(OTf)_2_), (**b**) [(L_2_-O)_2_Cu^II^_2_](ClO_4_)_2_·CH_3_CN (**3**_L2_-(ClO_4_)_2_·CH_3_CN) and (**c**) [(L_3_-O)_2_Cu^II^_2_](PF_6_)_2_ (**3**_L3_-(PF_6_)_2_) (only hydrogen atoms from NH moieties are shown and atoms coordinating to Cu are labelled for clarity).

Crystal structure for complex [(L_1_-O)_2_Cu^II^_2_](OTf)_2_ (**3**_L1_-(OTf)_2_) was obtained, and its ORTEP diagram is shown in Figure 3a. The molecule sits on a symmetrical center that transforms one macrocyclic ligand into the other. Each copper metal atom has a strongly distorted trigonal bipyramidal towards a square-planar pyramidal geometry (with a τ factor [38] of 0.56), and can be considered a mixture of both. Copper centers share coordinative sites with both ligands. Each copper atom is coordinated to a phenoxo O atom and an N atom from one of the macrocyclic ligands, and to the phenoxo O atom and two N atoms from the second macrocyclic ligand. The copper metal centers become doubly bridged by each macrocyclic ligand. The oxygen atoms of the phenoxo groups are bridging the copper metal centers so that the axial oxygen atom from one pyramid also occupies a position in the trigonal base of the other pyramid. The Cu_2_O_2_ core atoms lie in a plane forming a rhomboidal arrangement (Cu-O 1.930(2) Å, 2.174(2) Å), Cu···Cu 3.085 Å and O···O 2.718 Å).

Structures of complexes [(L_2_-O)_2_Cu^II^_2_](ClO_4_)_2_·CH_3_CN (**3**_L2_-(ClO_4_)_2_·CH_3_CN) and [(L_3_-O)_2_Cu^II^_2_](PF_6_)_2_ (**3**_L3_-(PF_6_)_2_) were also determined by X-ray diffraction (Figure 3b,c, respectively). Both dinuclear structures **3**_L2_ and **3**_L3_ bear the same ligand-donor set N_3_O_2_ per Cu atom and copper metal centers become doubly bridged by each macrocyclic ligand, as the previously described complex **3**_L1_. Each copper metal atom in complexes **3**_L2_ and **3**_L3_ has a strongly distorted trigonal bipyramidal towards a square-planar pyramidal geometry (with a τ factor of 0.61 for **3**_L2_ and 0.62 for **3**_L3_), featuring the same macrocyclic ligand size (14-membered). The oxygen atoms of the phenoxo groups in complex **3**_L2_ are bridging the copper metal centers so that the axial oxygen atom from one pyramid also occupies a position in the trigonal base of the other pyramid. The Cu_2_O_2_ core atoms lie in a plane forming a rhomboidal arrangement (Cu-O 1.925(1) Å, 2.128(2) Å, Cu···Cu 3.132 Å and O···O 2.581 Å). A rhomboidal arrangement of the Cu_2_O_2_ core is also found for complex **3**_L3_ (Cu-O 1.930(3) Å, 2.122(3) Å, Cu···Cu 3.103 Å and O···O 2.613 Å).

These structures are very rare, and to our knowledge there is only one precedent in the literature, reported in 2002 [39], where a small (12-membered) triazamacrocycle (L_4_-H, m = 2, n = 2, R_1_ = H) already showed the ability to form a bis-phenoxo-Cu^II^_2_ compound through route b (Figure 2), but no aryl-Cu^III^ was detected, probably due to its small size and its inability to accommodate aryl-Cu^III^ intermediate species. Contrary to the structures reported in this work, the smaller macrocycle favored a more square-planar geometry for each copper center (with a τ factor of 0.21).

The comparison of crystal structures of these complexes shows the same type of N_3_O_2_ coordination sphere for each Cu atom, although geometry environment for copper is directly related to conformational constraints imposed by ligand backbone. Thus, the trend found shows that the smaller size of the macrocycle favors square-pyramidal geometry (12-membered L_4_, τ factor of 0.21) [39], whereas 13-membered L_1_ afforded a τ factor of 0.56, and 14-membered macrocyclic rings (L_2_–L_3_) showed τ values in the range of 0.60–0.67.

### 2.3. Mechanistic Investigation on the Aromatic Hydroxylation Reaction

In order to gain more mechanistic insight of the C-O coupling by reaction of aryl-Cu^III^ with water under basic conditions, the synthetic conditions have been optimized for the synthesis [(L_2_-O)_2_Cu^II^_2_](ClO_4_)_2_ (**3**_L2_-(ClO_4_)_2_. In principle, any aqueous base reagent instead of KOH 1 M can be used to achieve the final product, as shown in Table 1. Interestingly, other *O*-containing reagents such as H_2_O_2_ are also able to perform the hydroxylation reaction. However, the addition of H_2_O_2_ 3% in water did not cause any change to copper(III) until the base Et_3_N was injected into the solution (see entries 6–7 in Table 1). From these series of reactions, it may be concluded that the addition of water or H_2_O_2_ does not affect the stability of the aryl-Cu^III^, and only the presence of a base triggers the reaction to bis-phenoxo complex formation through a colored intermediate. The presence of O_2_ in the solution in entry 5 was tested to check if it had any influence in reaction time-scale or final yield. No quenching of violet intermediate was found but differences in final yield were noticeable: 20% yield for reaction (entry 5) and 53% for entry 3. When using the hydrogen peroxide activated with DABCO (entries 8–9), we noticed that product **3**_L2_ was obtained in substantially better yield (40%, entry 9) when 0.5 equivalents of the DABCO.2H_2_O_2_ adduct were used.

**Figure 4:**
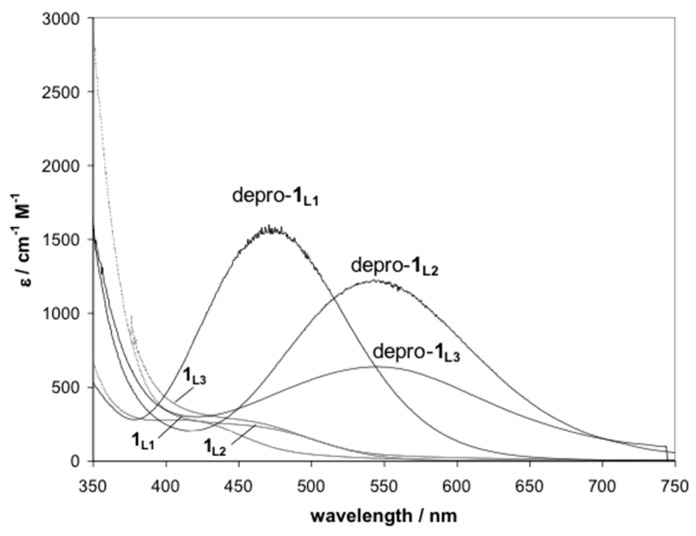
Electronic spectra of deprotonated aryl-Cu^III^ intermediates (depro-**1**_L1_, depro-**1**_L2_, depro-**1**_L3_) UV-Vis plots for aryl-Cu^III^ complexes ([(L_x_)Cu^III^]^2+^, x = 1–3) (**1**_L1_–**1**_L3_) are also shown in the plot.

The colored intermediate was characterized by UV-vis and corresponded to the deprotonated aryl-Cu^III^ species for L_1_–L_3_ systems (Figure 4), analogously to the reported case of deprotonated-**1**_L2_ complex (depro-**1**_L2_) [40]. In addition, weak axial coordination of a water molecule to the Cu^III^ center is proposed as a necessary species towards C-O reductive elimination. The same reactivity behavior is found for complex [(L_3_)Cu^III^]^2+^, whereas significant differences are shown by complex [(L_1_)Cu^III^]^2+^. For the latter, stability of red-brown intermediate depro-**1**_L1_ is much higher than for depro-**1**_L2_, depro-**1**_L3_, and reaction is not finished in less than 24 h upon KOH addition. In line with the enhanced stability, a significantly lower yield (30%) for the corresponding bis-phenoxo complex [(L_1_-O)_2_Cu^II^_2_]^2+^ (**3**_L1_) was found.

### 2.4. Aromatic Hydroxylation via Arene C-H Activation with Cu^I^/O_2_

The study of dioxygen activation by the Cu^I^ complexes synthesized with ligands L_1_–L_3_ demonstrated another mechanistic twist regarding formal aromatic C-H hydroxylations. Bubbling O_2_ to [(L_1_-H)Cu^I^](OTf) (**2**_L1-H_), [(L_2_-H)Cu^I^](OTf) (**2**_L2-H_), and [(L_3_-H)Cu^I^](OTf) (**2**_L3-H_) at room temperature in CH_3_CN caused the formation of intense colored intermediates resembling intermediates depro-**1**_L1_, depro-**1**_L2_, and depro-**1**_L3_, respectively (Figure 2, route b). Besides, decomposition of colored intermediates gives the same bis-phenoxo copper(II) complexes [(L_1_-O)_2_Cu^II^_2_]^2+^ (**3**_L1_), [(L_2_-O)_2_Cu^II^_2_]^2+^ (**3**_L2_) and [(L_3_-O)_2_Cu^II^_2_]^2+^ (**3**_L3_) as final products, although in significantly lower yields (25% isolated yield for **3**_L2_). The UV-vis monitoring of these reactions confirmed that hydroxylation was occurring through the same aryl-Cu^III^ intermediates, featuring the same LMCT bands in each case, albeit with lower intensities. In addition, ^1^H NMR monitoring of the O_2_ bubbling to [(L_1_-H)Cu^I^](OTf) (**2**_L1-H_) in CD_3_CN clearly shows the formation of peaks corresponding to depro-**1**_L1_ after 30 min (see Figure S1 in the Supplementary Materials), reaching full formation above 10 h [11,40]. The ESI-MS spectrum for violet intermediate obtained by reacting [(L_3_-H)Cu^I^]^+^ with O_2_ shows a characteristic peak at *m*/*z* = 294 corresponding to the fragment depro-**1**_L3_ (see Figure S2). Under these conditions, decomposition of the intermediate towards **3**_L2_ formation was slow and was detected after 40 h.

The lower yield obtained is related to the fact that the reaction of [(L_2_-H)Cu^I^](OTf) (**2**_L2-H_) with O_2_ first undergoes an oxidation to Cu^II^, which enables it to then undergo a C-H activation through a disproportionation reaction (50% aryl-Cu^III^ and 50% Cu^I^). Therefore, route b converges with route a (Figure 2) and the obtaining of the low 25% yield for **3**_L2_ through route b, compared to the 65% obtained through route a (Figure 2), mainly stems from the disproportionation pathway.

## 3. Materials and Methods

All reagents and solvents were purchased from Sigma Aldrich (Saint Louis, MO, USA) and used without further purification. Cu^III^ complexes [(L_x_)Cu^III^](X)_2_ (x = 1–3; X = ClO_4_^−^, OTf^−^, PF_6_^−^) [16,23], and Cu^I^ complexes [(L_1_-H)Cu^I^](OTf) (**2**_L1-H_), [(L_2_-H)Cu^I^](OTf) (**2**_L2-H_) and [(L_3_-H)Cu^I^](OTf) (**2**_L3-H_) were synthesized following reported procedures [41]. NMR data concerning product identity were collected with a Bruker 400 AVANCE (Billerica, MA, USA). Preparation and handling of air-sensitive Cu^I^ complexes were carried out in a N_2_ drybox. High resolution mass spectra (HRMS) were recorded on a Bruker MicrOTOF-Q IITM instrument (Billerica, MA, US) using ESI-MS at Serveis Tècnics University of Girona.

Warning: Although we have experienced no problems with the compounds reported herein, perchlorate salts are potentially explosive, and should only be handled in small quantities and never heated in a solid state.

[(L_1_-O)_2_Cu^II^_2_](OTf)_2_ (**3**_L1_-(OTf)_2_): the synthesis was carried out under N_2_. Into a solution of complex **1**_L1_-(OTf)_2_ (0.05 g, 8.7 × 10^−5^ mol) in CH_3_CN (1 mL) was injected a KOH(aq) 1M (87 μL, 8.7 × 10^−5^ mol). Reaction was stirred until the red-brown intermediate formed faded to green (48 h). Diffusion of diethyl ether and overnight storing at −25 °C allowed formation of green crystals in 30% isolated yield (0.012 g). ESI-MS (CH_3_CN): **3**_L1_743 [-(OTf)]^+^, 297 [(L_1_-O)Cu^II^]^+^; UV/Vis (CH_3_CN): λ_max_ (ε)= 394 (760), 699 (615); IR (KBr pellet, cm^−1^): 3258 (m), 3121 (m), 2924 (w), 1591 (w), 1456 (m), 1285 (s), 1252 (s), 1165 (m), 1031 (m), 640 (m); elemental analysis calcd for C_26_H_40_N_6_O_2_Cu_2_(C_2_F_6_S_2_O_6_)·0.5CH_3_CN (%): C 38.1, H 4.60, N 10.00, S 7.00; found: C 37.95, H 4.82, N 10.22, S 6.75.

Complex **3**_L1_-(OTf)_2_ can also be obtained by O_2_ bubbling of the Cu^I^ complex [(L_1_-H)Cu^I^](OTf) (**2**_L1-H_) (see synthesis of complex **3**_L2_).

[(L_2_-O)_2_Cu^II^_2_](OTf)_2_ (**3**_L2_-(OTf)_2_): Synthesis was carried out under N_2_. Into a solution of complex **1**_L2_-(OTf)_2_ (0.03 g, 4.9 × 10^−5^ mol) in CH_3_CN (2 mL) was injected a KOH(aq) 1M (50 μL, 5.0 × 10^−5^ mol). Reaction was stirred until the violet intermediate formed faded to green (2–3 h). Slow diffusion of diethyl ether allowed the formation of green crystals in 65% isolated yield (0.011 g). ESI-MS (CH_3_CN): 799 [**3**_L2_-(OTf)]^+^, 325 [(L_2_-O)Cu^II^]^+^; UV/Vis (CH_3_CN): λ_max_ (ε) = 411 nm (1000), 765 nm (610); IR (KBr pellet, cm^−1^): 3258 (m), 3210 (m), 2931 (m), 2869 (m), 1592 (m), 1463 (s), 1282 (s), 1236 (s), 1162 (s), 1022 (s), 636 (s); elemental analysis calcd for C_30_H_48_N_6_O_2_Cu_2_(C_2_F_6_S_2_O_6_) (%): C 40.50, H 5.10, N 8.80, S 6.70; found: C 40.10, H 5.40, N 8.40, S 6.40.

Perchlorate complex **3**_L2_-(ClO_4_)_2_ is synthesized in a similar manner, and X-ray quality crystals of **3**_L2_-(ClO_4_)_2_·CH_3_CN were obtained by recrystallization in CH_3_CN/ether.

Complex **3**_L2_-(OTf)_2_ can also be obtained by O_2_ bubbling of the Cu^I^ complex [(L_2_-H)Cu^I^](OTf) (**2**_L2-H_). Colorless complex [(L_2_-H)Cu^I^](OTf) (0.025 g, 5.4 × 10^−5^ mol) in 2 mL CH_3_CN/CH_2_Cl_2_ 1/3 under N_2_ is treated with 1.75 mL of dioxygen O_2_ (8.4 × 10^−5^ mol). Solution changes to violet slowly and after 3 h stirring fades to green. Slow diffusion of diethyl ether allowed isolation of bis-phenoxo complex **3**_L2_-(OTf)_2_ in 25% isolated yield.

[(L_3_-O)_2_Cu^II^_2_](OTf)_2_ (**3**_L3_-(OTf)_2_): synthesis was carried out under N_2_. Into a solution of complex **1**_L3_-(OTf)_2_ (0.03 g, 5.05 × 10^−5^ mol) in CH_3_CN (2 mL) was injected a KOH(aq) 1M (50 μL, 5.0 × 10^−5^ mol). Reaction was stirred until the violet intermediate formed faded to green (1–2 h). Slow diffusion of diethyl ether allowed the formation of green crystals in 60% isolated yield (0.014 g). ESI-MS (CH_3_CN): 771 [**3**_L3_-(OTf)]^+^, 311 [(L_3_-O)Cu^II^]^+^; UV/Vis (CH_3_CN): λ_max_ (ε) = 436 nm (830), 756 nm (485); IR (KBr pellet, cm^−1^): 3336 (m), 3294 (m), 1591 (m), 1465 (m), 1280 (s), 636 (s).

Complex **3**_L3_ can also be obtained from oxygenation of the corresponding Cu^I^ complex: colorless solution of complex [(L_3_-H)Cu^I^](PF_6_) (**2**_L1-H_) (0.025 g, 5.57 × 10^−5^ mol) in 2 mL CH_3_CN/CH_2_Cl_2_ 1/3 under Ar is treated with excess O_2_ at room temperature. Solution changes to violet and slowly and after 3 h stirring fades to green. Slow diffusion of diethyl ether allowed isolation of bis-phenoxo complex **3**_L3_-(PF_6_)_2_ in 20% isolated yield. ESI-MS (CH_3_CN): 767 [**3**_L3_-(PF_6_)]^+^, 311 [(L_3_-O)Cu^II^]^+^. Elemental analysis calcd for C_28_H_44_N_6_O_2_Cu_2_P_2_F_6_ (%): C 36.80, H 4.90, N 9.20; found: C 37.09, H 5.11, N 9.10.

X-ray diffraction analysis. The measurement was carried out on a BRUKER SMART APEX CCD diffractometer (Billerica, MA, US) using graphite-monochromated Mo Kα radiation (λ *=* 0.71073 Å). CCDC 2027155 (**3**_L1_-(OTf)_2_), 2027156 (**3**_L2_-(ClO_4_)_2_·CH_3_CN), 2027157 (**3**_L3_-(PF_6_)_2_) contain the supplementary crystallographic data for this paper.

## 4. Conclusions

In summary, seven well-defined aryl-Cu^III^ complexes featuring different ring sizes and different electronic properties have been reacted with water in basic conditions to produce intriguing bis-phenoxo-Cu^II^_2_ complexes (**3**_L1_–**3**_L6_), all of which are characterized by XRD. A structural trend correlating the size of the macrocycle and the geometry of each metal center is found, where the smaller 12-membered macrocycle ring (L_4_) favors square-pyramidal geometry, whereas 13-membered (L_1_) and 14-membered macrocyclic rings (L_2_–L_3_) favored trigonal bipyramidal geometries [39]. Mechanistic investigations indicate that the reaction takes place by an initial deprotonation of the NH group coordinated to Cu^III^ center, subsequent reductive elimination with H_2_O as nucleophile to form phenoxo products, and finally air oxidation of the Cu^I^ produced to form the final bis-phenoxo-Cu^II^_2_ complexes, whose enhanced stability acts as a thermodynamic sink and pushes the reaction forward. Furthermore, the corresponding [(L_x_-H)Cu^I^](OTf) (**2**_Lx-H_) complexes react with O_2_ to undergo 1e^−^ oxidation to Cu^II^ and subsequent C-H activation via disproportionation to form aryl-Cu^III^ species, which then undergo the same reaction path towards bis-phenoxo-Cu^II^_2_ complexes. Facile formation of bis-phenoxo-Cu^II^_2_ complexes through aryl-Cu^III^ reductive elimination with basic water is shown, and also the formal aromatic hydroxylation of arene substrates (L_x_-H) via aryl-Cu^III^ is mechanistically unraveled.

## Figures and Tables

**Table 1:** Different reactivity behavior of [(L_2_)Cu^III^](OTf)_2_ (**1**_L2_) in front of different bases and water content to finally obtain **3**_L2_. Typical experiment conditions: CH_3_CN, [Cu^III^] = 5–20 mM, N_2_ atmosphere (unless change specified), R.T. Traces of water are always present.

Entry	Reagents	Reaction Time (min)	Isolated Yield of 3_L2_ (%)
1	KOH (1 eq.), H_2_O (54 eq.)	60 min	65%
2	KOH (2 eq.), H_2_O (108 eq.)	25 min	8%
3	Proton Sponge (1 eq.), H_2_O (7 eq.)	180 min	53%
4	Proton Sponge (1 eq.)	60 min	0%
5	Proton Sponge (1 eq.), O_2_ (excess)	240 min	20%
6	H_2_O_2_ (3% in H_2_O) (1 eq.), H_2_O (52 eq.), Et_3_N (1 eq.)	10 min	31%
7	H_2_O_2_ (3% in H_2_O) (1 eq.), H_2_O (52 eq.)	60 min	0%
8	DABCO.2H_2_O_2_ (2 eq.)	45 min	15%
9	DABCO.2H_2_O_2_ (0.5 eq.)	40 min	40%

## Supplementary Materials

The following are available online, Figures S1 and S2. Figure S1: ^1^H NMR changes in [(L_1_-H)Cu^I^](OTf) (**2**_L1-H_) complex spectrum after O_2_ bubbling, Figure S2: ESI-MS of complex depro-**1**_L1_.
