# Supplementary material for: Bis-Phenoxo-CuII2 Complexes: Formal Aromatic Hydroxylation via Aryl-CuIII Intermediate Species
Source: Molecules. 2020 Oct 9;25(20):4595. doi: 10.3390/molecules25204595 (PMC7587178; doi:10.3390/molecules25204595)

molecules

MDPI

Supporting Information for

Bis-phenoxo-Cu^{II}_2 complexes: formal aromatic hydroxylation via Aryl-Cu^{III} intermediate species

Xavi Ribas*, Raül Xifra, Xavier Fontrodona

Figure S1. ^{1}H NMR changes in $[(L_1\text{-}H)Cu^I](OTf)$ ($\mathbf{2}_{L1\text{-}H}$) complex spectrum after O_2 bubbling in CD_3CN; a) as-synthesized Cu^I complex, b) 15 min reaction time, c) 40 min, d) 60 min, e) 130 min, f) 750 min.

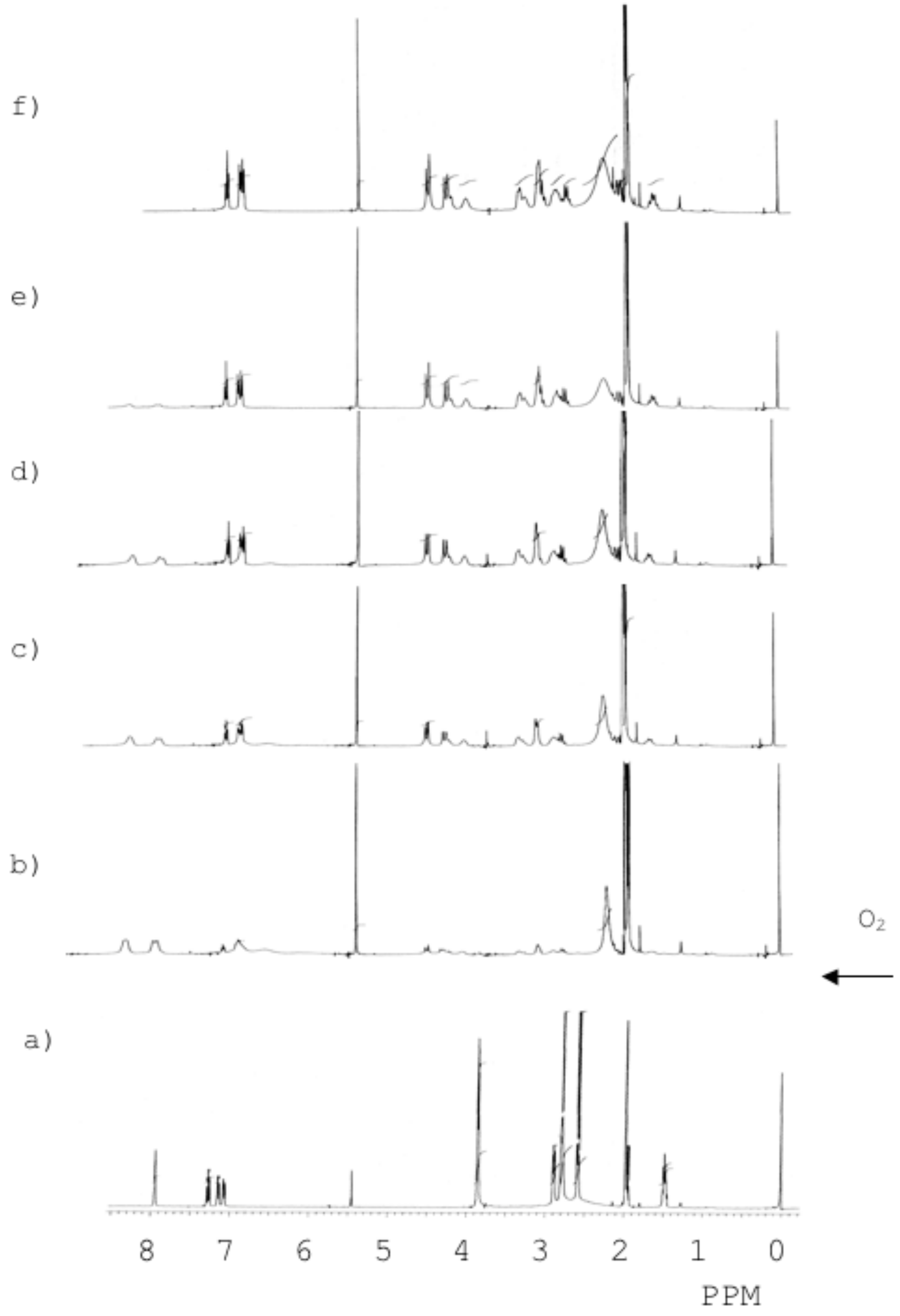

Figure S2. ESI-MS of complex depro-$\mathbf{1}_{L1}$ obtained by reaction of $[(L_1\text{-H})Cu^I]^+$ ($\mathbf{2}_{L1\text{-H}}$) + O_2 at R.T. in CH_3CN. Inset: simulated fragment peak.

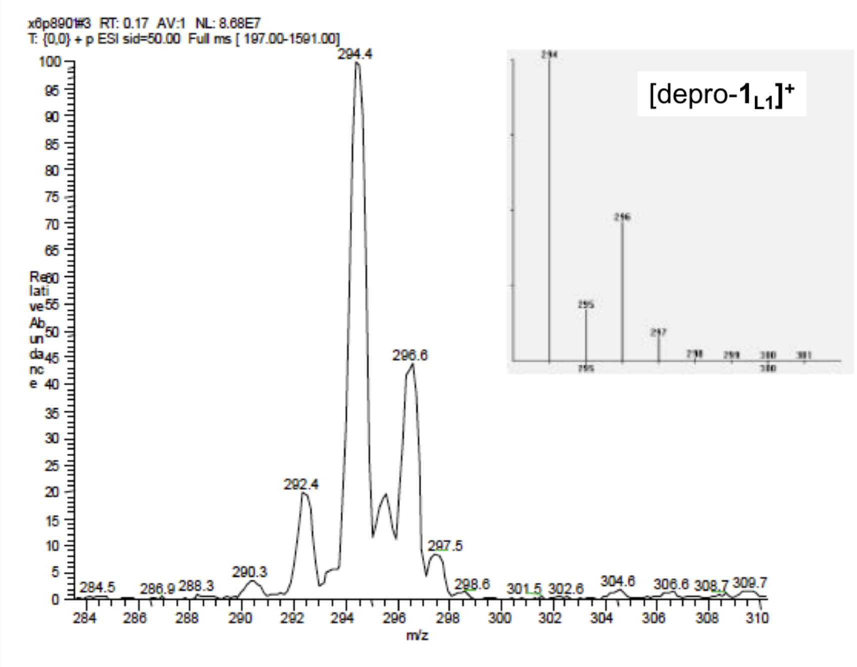

Supplement: Supplementary file 1 [file molecules-25-04595-s001.pdf]
